# Effects of physical exercise on the functionality of human nucleotidases: A systematic review

**DOI:** 10.14814/phy2.15464

**Published:** 2022-09-18

**Authors:** Cesar Eduardo Jacintho Moritz, Alexandra Ferreira Vieira, Denise de Melo‐Marins, Fabrício Figueiró, Ana Maria Oliveira Battastini, Alvaro Reischak‐Oliveira

**Affiliations:** ^1^ Programa de Pós‐Graduação em Ciências do Movimento Humano, Escola de Educação Física, Fisioterapia e Dança (ESEFID) Universidade Federal do Rio Grande do Sul (UFRGS) Porto Alegre Brazil; ^2^ Department of Applied Physiology and Kinesiology University of Florida Gainesville Florida USA; ^3^ Programa de Pós‐Graduação em Ciências Biológicas: Bioquímica Universidade Federal do Rio Grande do Sul (UFRGS) Porto Alegre Brazil; ^4^ Departamento do Bioquímica, Instituto de Ciências Básicas da Saúde (ICBS) Universidade Federal do Rio Grande do Sul (UFRGS) Porto Alegre Brazil

**Keywords:** 5′‐nucleotidase, exercise, NTPDase1, Nucleotidases

## Abstract

Nucleotidases contribute to the regulation of inflammation, coagulation, and cardiovascular activity. Exercise promotes biological adaptations, but its effects on nucleotidase activities and expression are unclear. The objective of this study was to review systematically the effects of exercise on nucleotidase functionality in healthy and unhealthy subjects. The MEDLINE, EMBASE, Cochrane Library, and Web of Science databases were searched to identify, randomized clinical trials, non‐randomized clinical trials, uncontrolled clinical trials, quasi‐experimental, pre‐, and post‐interventional studies that evaluated the effects of exercise on nucleotidases in humans, and was not limited by language and date. Two independent reviewers performed the study selection, data extraction, and assessment of risk of bias. Of the 203 articles identified, 12 were included in this review. Eight studies reported that acute exercise, in healthy and unhealthy subjects, elevated the activities or expression of nucleotidases. Four studies evaluated the effects of chronic training on nucleotidase activities in the platelets and lymphocytes of patients with metabolic syndrome, chronic kidney disease, and hypertension and found a decrease in nucleotidase activities in these conditions. Acute and chronic exercise was able to modify the blood plasma and serum levels of nucleotides and nucleosides. Our results suggest that short‐ and long‐term exercise modulate nucleotidase functionality. As such, purinergic signaling may represent a novel molecular adaptation in inflammatory, thrombotic, and vascular responses to exercise.

## Graphical abstract

Purinergic signaling represents a novel molecular adaptation in inflammatory, thrombotic, and vascular responses to the exercise. Acute and chronic protocols of exercise induce at least transitory adaptations in the functionality of human nucleotidases. The activity of nucleotidases may be linked to cardiometabolic parameters.

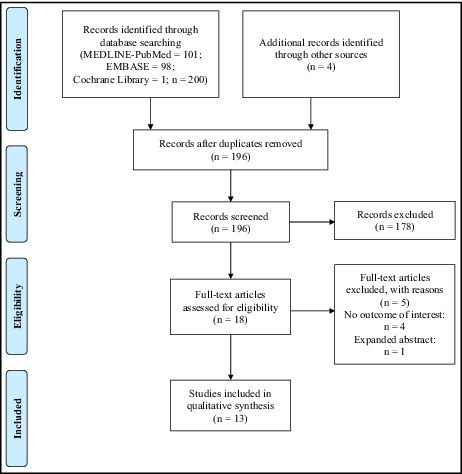

## 1 INTRODUCTION

Physical exercise is a trigger for multiple adaptations in physiological and biochemical systems, making exercise an important tool in the prevention and treatment of many diseases (Heinonen et al., 2014; Pedersen & Saltin, 2015). To elucidate the molecular and biochemical mechanisms involved in exercise, we decided to investigate purinergic signaling and its interaction with exercise physiology.

The concept of purinergic signaling is based on the existence of extracellular nucleotides and nucleosides, such as adenosine 5′‐triphosphate (ATP), adenosine 5′‐diphosphate (ADP), adenosine 5′‐monophosphate (AMP), and adenosine (ADO), which contribute to the modulation of immune, coagulation, cardiac, and vascular activity (Burnstock, 2017; Burnstock & Pelleg, 2015; Ralevic & Dunn, 2015). The source of these compounds in the extracellular environment occurs due to multiple pathways that include apoptosis, necrosis, cell injury, shear stress, exocytosis, ion channels, connexin, and pannexin channels (Lohman & Isakson, 2014; Yegutkin, 2014).

The effects triggered by purines and pyrimidines in the extracellular environment are mediated by the P1 and P2 purinergic receptors (Hechler & Gachet, 2015). The P1, or adenosine receptor, includes four subtypes: A_1_, A_2A_, A_2B,_ and A_3_, all of which are members of the G‐protein‐coupled family (Fredholm et al., 2011). P2 receptors are divided into P2X and P2Y; the P2X receptors are ionotropic, while P2Y are metabotropic receptors. ATP is the major agonist of P2X receptors, which has seven subtypes P2X (P2X_1–7_). The P2Y are also G‐protein‐coupled family receptors (P2Y_1_, P2Y_2_, P2Y_4_, P2Y_6_, P2Y_11_, P2Y_12_, P2Y_13,_ and P2Y_14_) and are selective to different nucleotides, such as ATP, ADP, uridine 5′‐triphosphate (UTP), and uridine 5′‐diphosphate (UDP) (Puchałowicz et al., 2014).

The purinergic system includes the enzymatic degradation of ATP and other compounds by nucleotidases, which control the levels of extracellular purines and pyrimidines and the magnitude of purinergic responses (Bagatini et al., 2018). The nucleotidase family includes nucleoside triphosphate diphosphohydrolases (NTPDases), nucleotide pyrophosphatases/phosphodiesterases (NPPs), and 5′‐nucleotidase/CD73 (5’‐NT) (Yegutkin, 2014). These enzymes can be found on the cell surface, attached to plasma membrane (Zimmermann et al., 2012), in their soluble forms in extracellular fluid or the bloodstream (Oses et al., 2004), and associated with microvesicles and exosomes (Jiang et al., 2014; Yegutkin, 2014).

The NTPDase family hydrolyzes extracellular nucleotide tri‐ and diphosphates, such as ATP and ADP, where the final product of NTPDase activity are monophosphates nucleotides. Eight members of the NTPDase family have been identified in mammalians (NTPDase1‐8) and these enzymes are expressed in almost every tissue (Robson et al., 2006; Zimmermann et al., 2012). The NPP family is composed of seven members (NPP1‐7) that hydrolyze pyrophosphate or phosphodiester bonds and have a great range of substrate specificity. The NPP1, NPP3, NPP4, and NPP5 enzymes hydrolyze nucleotides and/or dinucleotides (Lopez et al., 2020; Yegutkin, 2014). NTPDases and NPPs are co‐expressed in many cell types; thus, the catalytic properties of these enzyme families are complementary, but unlike hydrolysis by the NTPDases, ATP hydrolysis by NPPs results in direct conversion to AMP and pyrophosphate (PPi) as product(Yegutkin, 2008).

The ecto‐5’‐NT/CD73 enzyme (membrane‐anchored isoform), among other actions, catalyzes the formation of extracellular ADO via AMP hydrolysis, shutting down ATP and ADP signaling pathways by P2 receptors. The formation of extracellular ADO allows P1 receptors activation, triggering their signaling pathways (Yegutkin, 2008; Zimmermann et al., 2012). The purinergic cascade proceeds with adenosine deamination and inosine (INO) as a product. In this context, adenosine deaminase (ADA) is an important enzyme, being mainly responsible for extracellular inosine levels and ADO metabolism. Additionally, nucleoside transporters can reuptake these nucleosides into cells to reestablish intracellular levels of ATP (Antonioli et al., 2012; Haskó et al., 2004; Yegutkin, 2014).

Some animal model studies have shown the relationship between exercise and nucleotidase functionality. Langfort et al. investigated the effects of 6 weeks of endurance and sprint training on ADA and 5’‐NT/CD73 activities in the rat heart, demonstrating an increase in 5’‐NT/CD73 basal activity after both training modalities (Langfort et al., 1996). Roque et al. evaluated the effects of swimming training for 6 weeks on rat serum and cardiac nucleotidase functionality; the authors found an increase in ATP, ADP, and AMP hydrolysis in the blood serum and cardiac sarcolemma. Besides that, swimming upregulated of NTPDase1/CD39 and 5’‐NT/CD73 in the rat heart (Roque et al., 2011). Cardoso et al. investigated the effects of 6 weeks of swimming training on nucleotidase activities in the platelets of hypertensive rats and demonstrated that 6 weeks of this exercise protocol prevented the increase in platelet nucleotidase activities that was promoted by the hypertensive state (Cardoso et al., 2012).

In recent years, few studies have focused on investigating the influence of physical exercise on nucleotidase activities and expression in humans. Previous studies present a large methodological variety, small sample sizes, as well as a heterogeneity of exercise protocols and populations studied (Coppola et al., 2005; Karabulut et al., 2011; Moritz et al., 2017; Yegutkin et al., 2007). Despite the current data, there is a lack of knowledge about the role of purinergic signaling components in acute and chronic adaptations promoted by physical exercise in different populations. The understanding of these mechanisms may allow us the development of further therapeutic strategies focused on health and performance improvements. The aim of the present study was to review systematically the effects of acute, chronic, aerobic, and anaerobic exercise on nucleotidase activities and expression in healthy and unhealthy subjects. Therefore, we aim to provide what we believe is the first systematic review of the effects of exercise on purinergic signaling to improve knowledge regarding exercise biochemistry.

## 2 METHODS

The present systematic review was conducted in accordance with the *Preferred Reporting Items for Systematic Review and Meta‐Analyses* (PRISMA) statement (Liberati et al., 2009) and was registered on the *International Prospective Register of Systematic Reviews* (PROSPERO) under registration number CRD42019110593.

### 2.1 Eligibility criteria

For this study, the PICOS (Population, Intervention, Comparator, Outcomes, Study Design) was the following to identify the inclusion criteria: (1) Population: studies with healthy or unhealthy subjects, adults (≥20 years old) or elderly persons (≥65 years old), male or female gender were included; (2) Intervention: were included studies that evaluated acute, chronic, aerobic, or anaerobic exercise modalities; (3) Comparator: pre‐exercise period or untrained controls; (4) Outcomes: the primary outcomes for this study were nucleotidase activities and/or expression (e.g., NTPDase1/CD39 and 5′‐nucleotidase/CD73). Moreover, the secondary outcomes were the concentration of nucleotides and nucleosides, such as ATP, ADP, and ADO; (5) Study design: were included randomized clinical trials (RCT), non‐randomized clinical trials (non‐RCT), uncontrolled clinical trials, quasi‐experimental, and pre‐ and post‐intervention studies.

### 2.2 Search strategy

The search strategy was conducted in the following electronic databases: MEDLINE (via PubMed), EMBASE, and Cochrane Library. Moreover, a manual search of the references cited in published studies was performed. The search was carried out in February 2022 and was not limited by language or date and comprised the following terms and corresponding synonyms: “Exercise”, “Nucleotidases”, “Ectonucleotidases”, “NTPDases”, “E‐NTPDases”, “NTPDase1”, “E‐NTPDase1”, “CD39”, “5’‐Nucleotidase”, “Ecto‐5′‐nucleotidase”, “CD73”, “E‐NPP”, “NPP”, “Adenosine Deaminase”, “ATP”, “ADP”, “AMP”, “Adenosine”, “Inosine”. The complete search strategy used in PubMed is described in Table S1.

### 2.3 Study selection

Two investigators (A.F.V. and D.M.M.) independently evaluated titles and abstracts from studies found by the search strategy. When abstracts did not provide sufficient data about inclusion and exclusion criteria, the complete article was evaluated. In the second phase of study selection, full‐texts were evaluated and selected by the reviewers independently. The selection of studies was based on accordance with the eligibility criteria. Disagreements were settled by consensus, in cases of continuing disagreement a third investigator (C.E.J.M.) was consulted.

### 2.4 Data extraction

Two reviewers (A.F.V. and D.M.M.) performed data extraction independently, using standardized forms through Microsoft Excel® software. Methodological features of selected studies were collected, such as authors, year of publication, sample, methods, intervention, and results. For data that were presented only graphically, the results were extracted using WebPlotDigitizer (Rohatgi, 2021). In cases of disagreements, a third investigator was consulted to provide a consensus opinion (C.E.J.M.). The major outcomes extracted were nucleotidase activities and/or expression in the biological sample evaluated (e.g., serum, plasma, platelets, or lymphocytes).

### 2.5 Assessment of risk of bias

The assessment of the methodological quality of studies included was performed by two reviewers (A.F.V. and D.M.M.) independently, using the Downs and Black instrument (Downs & Black, 1998). This tool is a validated scoring system used to assess the methodological quality of randomized and non‐randomized studies; higher scores indicate less risk of bias, whereas lower scores indicate a higher risk of bias. The maximum score is 32 points, obtained through five domains: (1) reporting, 10 items (11 points); (2) external validity, 3 items (3 points); (3) internal validity—bias assessment, 7 items (7 points); (4) internal validity—confounding assessment, 6 items (6 points); (5) power, item (5 points). In cases of disagreements between the reviewers, a third investigator was consulted for a consensus (C.E.J.M.).

### 2.6 Data analysis

Data analysis was performed as a descriptive and qualitative analysis. Due to the methodological heterogeneity of studies included, it was impossible to perform meta‐analyses. The descriptive and qualitative data analyzed are presented in the figures and tables.

## 3 RESULTS

### 3.1 Study selection

**FIGURE 1:**
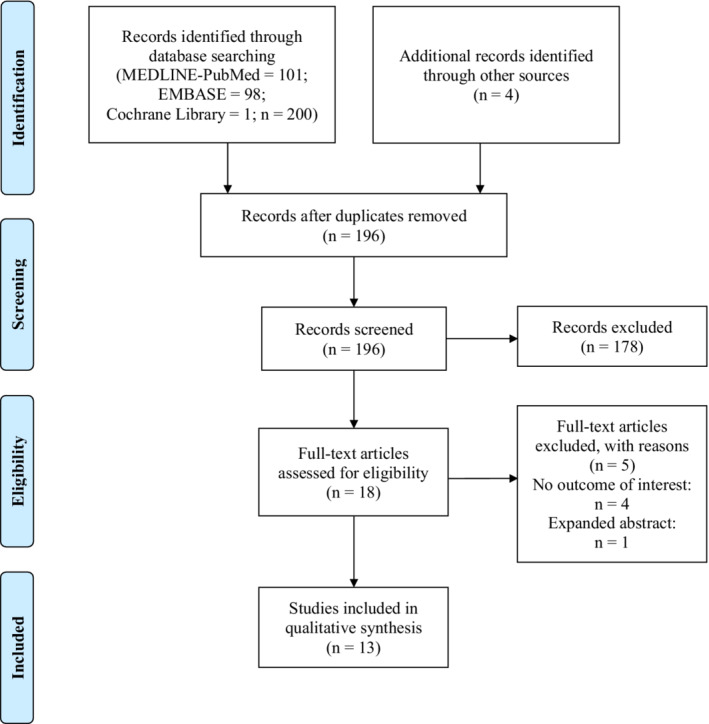
Flowchart of the included studies.

The initial search located 204 studies; eight studies were excluded because they were duplicated among the databases searched. Despite we did not include any language limitation, our search strategy only found studies in English. Of the remaining 196 studies, 178 were excluded based on their titles and/or abstracts. After full‐text analysis, five articles were excluded; four did not address the outcome of interest and the other was an expanded abstract. After full‐text evaluation, 13 studies were included in this review (Coppola et al., 2005; da Silveira et al., 2018; Dorneles et al., 2019; Karabulut et al., 2011; Kirby et al., 2012; Lammers et al., 2020; Mânica et al., 2020; Martins, Bagatini, Cardoso, Zanini, Abdalla, Baldissarelli, Dalenogare, dos Santos, et al., 2016; Martins, Bagatini, Cardoso, Zanini, Abdalla, Baldissarelli, Dalenogare, Farinha, et al., 2016; Miron et al., 2019; Moritz et al., 2017; Moritz et al., 2021; Yegutkin et al., 2007) (Figure 1).

### 3.2 Description of studies

The main characteristics of the included studies are shown in Table 1. Among the 13 included studies, eight (61.53%) (Coppola et al., 2005; Dorneles et al., 2019; Karabulut et al., 2011; Kirby et al., 2012; Miron et al., 2019; Moritz et al., 2017; Moritz et al., 2021; Yegutkin et al., 2007) exclusively studied healthy individuals and five (38.47%) (da Silveira et al., 2018; Lammers et al., 2020; Mânica et al., 2020; Martins, Bagatini, Cardoso, Zanini, Abdalla, Baldissarelli, Dalenogare, dos Santos, et al., 2016; Martins, Bagatini, Cardoso, Zanini, Abdalla, Baldissarelli, Dalenogare, Farinha, et al., 2016) reported on individuals with a disease. With the regard to the studies with healthy individuals, two studies (15.38%) (Karabulut et al., 2011; Moritz et al., 2017) included healthy and sedentary subjects, one (7.7%) (Kirby et al., 2012) included healthy young and older adults, one (7.7%) (Miron et al., 2019) included semi‐professional athletes and four (30.76%) (Coppola et al., 2005; Dorneles et al., 2019; Moritz et al., 2021; Yegutkin et al., 2007) studies included healthy sedentary and trained/physically active subjects. With respect to studies that included unhealthy individuals, two studies (15.38%) (Martins, Bagatini, Cardoso, Zanini, Abdalla, Baldissarelli, Dalenogare, dos Santos, et al., 2016; Martins, Bagatini, Cardoso, Zanini, Abdalla, Baldissarelli, Dalenogare, Farinha, et al., 2016) included metabolic syndrome patients and healthy individuals, one (7.7%) (da Silveira et al., 2018) included chronic kidney disease patients and two (15.38%) (Lammers et al., 2020; Mânica et al., 2020) included hypertensive patients. A total of 447 subjects were included in this systematic review, 274 (61.30%) male and 173 (38.70%) female subjects, with a mean age of 39.05 years (variation 20.56–67.2 years).

**TABLE 1:** Sample population characteristics of included studies.

Study, year, country	Characteristics of subjects	Sample size (M/F)	Age (years)	Body mass (kg)	BMI (kg/m^2^)
Coppola et al., 2005, Italy	G1: Healthy, sedentary G2: Healthy, physically active	G1: 08 (M) G2: 08 (M)	G1: 34 ± 7 G2: 34 ± 6	Not informed	G1: 26.3 ± 3 G2: 26.7 ± 3
Yegutkin et al., 2007, ^a^, Finland	Healthy, endurance‐trained	20 (M)	29 ± 3	78.9 ± 8.2	24.4 ± 2.5
Yegutkin et al., 2007, ^a^, Finland	Healthy, sedentary	7 (3 M/04 F)	30 ± 5	80.6 ± 23.1	27.9 ± 8
Karabulut et al., 2011, Turkey	G1: Healthy, sedentary males G2: Healthy, sedentary females	G1: 20 (M) G2: 20 (F)	G1: 21.67 ± 0.69 G2: 20.56 ± 0.75	G1: 76.42 ± 8.94 G2: 61.32 ± 6.76	G1: 23.6 ± 2.8 G2: 21.1 ± 2.3
Kirby et al., 2012, USA	G1: Healthy, young adults G2: Healthy, older adults	G1: 38 (33 M/05 F) G2: 26 (24 M/02 F)	G1: 23 ± 6.16 G2: 64 ± 5	Not informed	G1: 23.7 ± 1.84 G2: 26.5 ± 3.56
Martins, Bagatini, Cardoso, Zanini, Abdalla, Baldissarelli, Dalenogare, Farinha, et al., 2016, Brazil	G1: Metabolic syndrome patients G2: Healthy individuals	G1: 38 (15 M/23 F) G2: 30 (13 M/17 F)	G1: 59.4 ± 3.0 G2: 58.3 ± 2.8	Not informed	G1: 35.13 ± 5.13 G2: 23.2 ± 3.3
Martins, Bagatini, Cardoso, Zanini, Abdalla, Baldissarelli, Dalenogare, dos Santos, et al., 2016, Brazil	G1: Metabolic syndrome patients G2: Healthy individuals	G1: 20 (15 M/05 F) G2: 20 (14 M/06 F)	G1: 57.95 ± 3.30 G2: 59.40 ± 3.95	G1: 95.25 ± 9.91 G2: 74.30 ± 11.41	G1: 38.24 ± 8.69 G2: 23.93 ± 2.88
Moritz et al., 2017, Brazil	Healthy, sedentary	10 (M)	25.30 ± 2.94	81.51 ± 13.43	25.59 ± 3.44
Miron et al., 2019, Brazil	Semi‐professional athletes	19 (M)	27 ± 2.2	75 ± 8.6	21.4 ± 9.7
da Silveira et al., 2018, Brazil	Chronic kidney disease hemodialysis patients	34 (18 M/16 F)	50.95 ± 18.4	Not informed	Not informed
Dorneles et al., 2019, Brazil	G1: Low fitness, healthy G2: High fitness, healthy	G1: 15 (M) G2: 15 (M)	G1: 25.3 ± 1.4 G2: 26.1 ± 1.9	G1: 70.8 ± 3.68 G2: 71.3 ± 2.20	G1: 23.5 ± 1.5 G2: 23.7 ± 1.3
Lammers et al., 2020, Brazil	G1: Hypertensive patients, sedentary G2: Normotensive individuals, sedentary	G1: 31 (F) G2: 28 (F)	G1: 56.17 ± 4.3 G2: 53.64 ± 3.6	G1: 71.21 ± 3.5 G2: 66.61 ± 2.6	G1: 29.85 ± 4.9 G2: 25.22 ± 4.5
Mânica et al., 2020, Brazil	Hypertensive patients, sedentary	16 (F)	67.2 ± 3.7	70.7 ± 8.1	29.2 ± 3.7
Moritz et al., 2021, Brazil	G1: Normal weight sedentary G2: Overweight sedentary G3: Normal weight physically active	G1: 08 (M) G2: 08 (M) G3: 08 (M)	G1: 26.37 ± 2.97 G2: 25.75 ± 2.91 G3: 23.12 ± 3.18	G1: 74.16 ± 9.74 G2: 85.02 ± 5.95 G3: 75.01 ± 7.63	G1: 23.40 ± 2.01 G2: 25.75 ± 1.26 G3: 23.65 ± 1.2

*Note*: Data presented as mean ± standard deviation (SD).

Abbreviations: F, female; G, group; BMI, body mass index; M, male; NYHA, New York Heart Association.

^a^ Two studies are presented in the same article.

Descriptions of the exercise protocols applied in the selected studies are provided in Table 2. Nine (69.23%) (Coppola et al., 2005; Dorneles et al., 2019; Karabulut et al., 2011; Kirby et al., 2012; Mânica et al., 2020; Miron et al., 2019; Moritz et al., 2017; Moritz et al., 2021; Yegutkin et al., 2007) studies investigated the acute responses of exercise on the activity and/or expression of nucleotidases, four (30.77%) (da Silveira et al., 2018; Lammers et al., 2020; Martins, Bagatini, Cardoso, Zanini, Abdalla, Baldissarelli, Dalenogare, dos Santos, et al., 2016; Martins, Bagatini, Cardoso, Zanini, Abdalla, Baldissarelli, Dalenogare, Farinha, et al., 2016) studies focused on the effects of chronic exercise on nucleotidase behavior. Among the studies that applied acute protocols of exercise, two (15.38%) (Coppola et al., 2005; Yegutkin et al., 2007) used an incremental test, one (7.69%) (Karabulut et al., 2011) used the 20‐meter shuttle run test, two (7.69%) (Moritz et al., 2017; Moritz et al., 2021) employed constant intensity aerobic exercise, two (15.38%) (Dorneles et al., 2019; Miron et al., 2019) used high‐intensity interval exercise (HIIE), one (7.69%) (Kirby et al., 2012) used resistance handgrip exercise and one (7.69%) (Mânica et al., 2020) used low‐intensity aerobic exercise with blood flow restriction. Additionally, of studies that employed protocols of chronic exercise, two (15.38%) (Martins, Bagatini, Cardoso, Zanini, Abdalla, Baldissarelli, Dalenogare, dos Santos, et al., 2016; Martins, Bagatini, Cardoso, Zanini, Abdalla, Baldissarelli, Dalenogare, Farinha, et al., 2016) used concurrent training for 30 weeks and two (15.38%) (da Silveira et al., 2018; Lammers et al., 2020) used resistance training for 8 and 27 weeks, respectively.

**TABLE 2:** Main results summary of included studies.

Study, year, country	Exercise protocol	Sample analyzed	Results
Coppola et al., 2005, Italy	Incremental test: the test started with the workload of 30 W and load increments of 10 W/min. The test was finished at exhaustion.	Platelet B‐Lymphocytes T‐Lymphocytes	↓ CD39 expression in platelets post‐exercise (G1, 2‐fold; G2, 1.71‐fold). ↑ CD39 expression B‐Lymphocytes post‐exercise (G1, 2‐fold; G2, 1.28‐fold). ↔ in CD39 expression in T‐Lymphocytes post‐exercise.
Yegutkin et al., 2007, ^a^, Finland	Protocol 1: 15 min of submaximal exercise on a cycle ergometer, followed by 3 min rest and a constant maximal load exercise bout. Protocol 2: Incremental test in cycle ergometer with the workload of 25, 50, 75, 90, and 100% of peak power until exhaustion.	Blood plasma Blood serum	Protocol 1: ↑ ATP and ADP levels at submaximal (ATP, 1.68‐fold; ADP, 1.82‐fold) and maximal (ATP, 2.43‐fold; ADP, 2.37‐fold) exercise. ↑ AMP levels during maximal exercise (2.‐fold). ↑ NPP and NTPDase activities during submaximal (NPP, 1.29‐fold; NTPDase, 1.31‐fold) and maximal (NPP, 1.18‐fold; NTPDase 1.69‐fold) exercise. Protocol 2: ↑ NTPDase activity during exercise in venous (1.31‐fold) and arterial (1.71‐fold) blood.
Yegutkin et al., 2007, ^a^, Finland	Incremental cycle‐ergometer exercise to exhaustion.	Blood plasma	↑ ATP levels during maximal exercise (1.45‐fold). ↑ NTPDase (1.31‐fold) and NPP (1.21‐fold) activities during maximal exercise.
Karabulut et al., 2011, Turkey	Twenty‐meter shuttle run.	Blood plasma	↔ in ADA activity post‐exercise (G1). ↑ activity of ADA post‐exercise (G2, 13.53‐fold).
Kirby et al., 2012, USA	Fifteen minutes of graded‐intensity handgrip exercise: 5 min period each of 5, 15, and 25% of MVC workload.	Whole blood Blood plasma	↑ ATP hydrolysis at 5 (2.10‐fold), 15 (2.15‐fold), and 25% (2.21‐fold) of MVC (G1 and G2). ↑ levels of ATP at 5 (1.54‐fold), 15 (1.69‐fold), and 25% (2.03‐fold) of MVC (G1)
Martins, Bagatini, Cardoso, Zanini, Abdalla, Baldissarelli, Dalenogare, Farinha, et al., 2016, Brazil	Concurrent and moderate training with aerobic and resistance exercises, during 30 weeks/three times per week.	Platelets	↑ NTPDase (ATP, 2.41‐fold; ADP, 1.55‐fold), E‐5′‐nucleotidase (2.19‐fold), and NPP (6.97‐fold) activities pre‐training (G1). ↓ NTPDase (ATP, 1.86‐fold; ADP, 1.44‐fold), E‐5′‐nucleotidase (1.59‐fold), and NPP (4.66‐fold) activities post‐training (G1). ↓ ADA (2.1‐fold) activity pre‐training (G1). ↑ ADA (1.92‐fold) activity post‐training (G1).
Martins, Bagatini, Cardoso, Zanini, Abdalla, Baldissarelli, Dalenogare, dos Santos, et al., 2016, Brazil	Concurrent and moderate training with aerobic and resistance exercises, during 30 weeks/three times per week.	Lymphocytes	↑ NTPDase (ATP, 1.67‐fold; ADP, 2.11‐fold) activity pre‐training (G1). ↓ NTPDase (ATP, 1.54‐fold; ADP, 2.07‐fold) activity post‐training (G1). ↓ ADA (1.53‐fold) activity pre‐training (G1). ↑ ADA (1.64‐fold) activity post‐training (G1).
Moritz et al., 2017, Brazil	Thirty minutes of aerobic exercise on treadmill at 70% of MHR.	Blood serum	↑ ATP (2.62‐fold), ADP (2.59‐fold), AMP (1.53‐fold), and *p*‐Nph‐5’‐TMP (1.48‐fold) hydrolysis post‐exercise. ↓ levels of ATP (1.27‐fold) and ADP (1.22‐fold) post‐exercise; ↑ levels of ADO (1.23‐fold), INO (1.24‐fold), and UA (1.24‐fold) post‐exercise.
Miron et al., 2019, Brazil	HIIE: 0 to 10 min at 55% of MHR; 10 to 20 min at >90% of MHR; 20 to 30 min at 50–70% of MHR; 30 to 40 min at >90% of MHR.	Platelets Lymphocytes	↔ ATP, ADP, and AMP hydrolysis post‐exercise (Lymphocytes). ↔ NTPDase1 expression post‐exercise (Lymphocytes). ↑ ADA (2.28‐fold) activity post‐exercise (Lymphocytes). ↓ ATP (1.28‐fold) and ADP (1.44‐fold) hydrolysis post‐exercise (Platelets). ↑ ADA (1.83‐fold) activity post‐exercise (Platelets).
da Silveira et al., 2018, Brazil	Resistance training for 8 weeks/three times per week. Subjects performed three sets of 12–15 repetitions, with 1–2 kg.	Platelets	↓ ATP (2.11) and AMP (1.61‐fold) hydrolysis post‐training. ↔ ADP hydrolysis and ADO deamination post‐training.
Dorneles et al., 2019, Brazil	High‐intensity interval exercise, consisted of 10 bounts of 60 sec at 85–90% of MHR, alternated with 75 sec at 50 MHR in treadmill.	PBMC	↑ CD39 Expression post‐exercise on CD4^+^CD25^+^ (G1, immediately post 1.34‐fold, post‐1 h 1.3‐fold; G2, immediately post 1.2‐fold, post‐1 h 1.33‐fold) and CD4^+^CD25^−^ (G1, immediately post 2.03‐fold, post 1 h 1.58‐fold; G2, immediately post 1.38‐fold) T cells. ↑ CD73 expression post‐exercise on CD4^+^CD25^+^ T cells (G1, immediately post 1.6‐fold, post‐1 h 1.42‐fold; G2, immediately post 1.3‐fold, post‐1 h 1.36‐fold). ↔ CD73 expression post‐exercise on CD4^+^CD25^−^ T cells.
Lammers et al., 2020, Brazil	Resistance training for 27 weeks/two times per week/45–60 min of continuous exercise per day, moderate intensity.	Lymphocytes Blood serum	↓ ATP (G1, 1.25‐fold), and ADP (G1, 1.33‐fold) hydrolysis post‐training. ↓ ADA (G1, 1.28‐fold) activity post‐training. ↔ NTPDase1 and 2 expression post‐training. ↓ levels of ATP (G1, 1.17‐fold) post‐training.
Mânica et al., 2020, Brazil	Protocol 1: high‐intensity aerobic exercise, 50% of VO_2max_ for 10 min. Protocol 2: low‐intensity aerobic exercise, 30% of VO_2max_ for 10 min. Protocol 3: low‐intensity aerobic exercise with blood flow restriction, 30% of VO_2max_ and occlusion pressure to 130% of systolic blood pressure at rest.	Lymphocytes	Protocol 1: ↑ ATP (1.30‐ fold), ADP (1.18‐fold) hydrolysis, and ↓ADA (1.84‐fold) activity 30 min after exercise. Protocol 2: ↔ ATP, ADP hydrolysis, and ADA activity post‐exercise. Protocol 3: ↑ ATP (1.44‐fold), ADP (1.16‐fold) hydrolysis, and ↔ ADA activity 30 min after exercise.
Moritz et al., 2021, Brazil	Thirty minutes of aerobic exercise on treadmill at 70% of VO_2peak_.	Blood plasma	↑ ATP hydrolysis post‐exercise (G1, immediately post 1.66‐fold; G2, immediately post 1.90‐fold; G3, immediately post 1.61‐fold, post‐1 h 1.32‐fold). ↑ ADP hydrolysis post‐exercise (G1, immediately post 1.61‐fold; G2, immediately post 1.67‐fold, post‐1 h 1.36‐fold; G3, immediately post 1.60‐fold). ↑ AMP hydrolysis post‐exercise (G1, immediately post 1.55‐fold, post 1‐h 1.38‐fold; G2, immediately post 1.74‐fold, post‐1 h 1.61‐fold; G3, immediately post 1.61‐fold; 1.26‐fold post‐1 h). ↓ levels of ATP (G1, immediately post 1.45‐fold, post‐1 h 1.23‐fold; G2, immediately post 1.36‐fold, post‐1 h 1.25‐fold; G3, immediately post 1.33‐fold, post‐1 h 1.36‐fold). ↓ levels of ADP (G1, immediately post 1.60‐fold, post‐1 h 1.36‐fold; G2, immediately post 1.42‐fold, post‐1 h 1.23‐fold; G3, immediately post 1.47‐fold, post‐1 h 1.15‐fold). ↑ levels of ADO (G1, immediately post 1.17‐fold, post‐1 h 1.14‐fold; G2, immediately post 1.10‐fold, post‐1 h 1.20‐fold; G3, immediately post 1.14‐fold, post‐1 h 1.15‐fold). ↑ levels of INO (G1, immediately post 1.87‐fold; G2, immediately post 1.57‐fold, post‐1 h 1.47‐fold, G3, immediately post 1.5‐fold). ↑ levels of UA (G1, immediately post 1.33‐fold, post‐1 h 1.27‐fold; G2, immediately post 1.28‐fold, post‐1 h 1.37‐fold; G3, immediately post 1.16‐fold).

*Note*: Data presented as mean ± standard deviation (SD).

Abbreviations: ↑, significant increase; ↓, significant decrease; ↔, no change; ADA, adenosine deaminase; ADO, adenosine; ADP, adenosine 5′’‐diphosphate; AMP, adenosine 5′’‐monophosphate; ATP, adenosine 5′’‐triphosphate; BP, blood pressure; G, group; HIIE, high‐intensity intermittent training; HX, hypoxanthine; INO, inosine; MHR, maximum heart rate; MVC, maximum voluntary contraction; NPP, nucleotide pyrophosphatase/phosphodiesterase; NTPDase, nucleoside triphosphate diphosphohydrolases; PBMC, peripheral blood mononuclear cells; *p*‐Nph‐5’‐TMP, *p*‐nitrophenyl 5′’‐thymidine monophosphate; RPM, rotations per minute; UA, uric acid; VO_2max_, maximum oxygen uptake; VO_2peak_, peak oxygen uptake.W, watt.

^a^ Two studies are presented in the same article.

### 3.3 Assessment of methodological quality

The methodological quality assessment of the studies included was performed using the Downs and Black instrument (Downs & Black, 1998) and the results are presented in Table 3. The mean score for the included studies was 18.3/32 (57.21% of total, variation 16–23). The three majors' sources of bias were: (1) attempt to blind the study subjects (item 14); (2) attempt to blind those measuring the main outcomes (item 15); (3) randomization assignment concealed from patients and staff until recruitment was complete (item 24). Moreover, it is relevant to mention, that in studies where exercise is the main intervention, is not possible blind the subjects from studies. Therefore, in fact, item 14 from this assessment instrument is not applicable.

**TABLE 3:** Methodological quality of included studies, according to Downs and Black instrument (1998).

Study, year	Items																											
	1	2	3	4	5	6	7	8	9	10	11	12	13	14	15	16	17	18	19	20	21	22	23	24	25	26	27	Score
Coppola et al., 2005	1	1	1	1	2	1	1	0	1	0	0	0	1	0	0	1	1	1	1	1	0	0	0	0	0	1	0	16/32
Yegutkin et al., 2007	1	1	1	1	2	1	1	0	1	0	0	0	1	0	0	1	1	1	1	1	0	0	0	0	0	1	0	16/32
Karabulut et al., 2011	1	1	1	1	1	1	1	0	1	1	1	0	0	0	0	1	1	0	1	1	1	1	0	0	0	1	0	17/32
Kirby et al., 2012	1	1	0	1	2	1	1	0	1	0	0	0	1	0	0	1	1	1	1	1	0	0	0	0	1	1	0	16/32
Martins, Bagatini, Cardoso, Zanini, Abdalla, Baldissarelli, Dalenogare, Farinha, et al., 2016	1	1	1	1	2	1	1	0	1	1	1	0	1	0	0	1	1	1	1	1	1	0	0	0	1	1	0	20/32
Martins, Bagatini, Cardoso, Zanini, Abdalla, Baldissarelli, Dalenogare, dos Santos, et al., 2016	1	1	1	1	2	1	1	0	1	1	1	0	1	0	0	1	1	1	1	1	1	0	0	0	1	1	0	20/32
Moritz et al., 2017	1	1	1	1	2	1	1	0	1	0	0	0	1	0	0	1	1	1	1	1	0	0	0	0	0	1	0	16/32
Miron et al., 2019	1	1	1	1	2	1	1	0	1	0	1	0	1	0	0	1	1	1	1	1	1	1	0	0	0	1	0	19/32
da Silveira et al., 2018	1	1	1	1	1	1	1	0	1	1	0	0	1	0	0	1	1	1	1	1	1	0	0	0	1	1	0	18/32
Dorneles et al., 2019	1	1	1	1	2	1	1	0	1	1	0	0	1	0	0	1	1	1	1	1	1	0	0	0	0	1	0	18/32
Lammers et al., 2020	1	1	1	1	2	1	1	0	1	0	1	0	1	0	0	1	1	1	1	1	1	0	0	0	1	1	4	23/32
Mânica et al., 2020	1	1	0	1	1	1	1	1	1	0	1	1	1	0	0	1	1	1	1	1	1	0	1	0	0	1	1	20/32
Moritz et al., 2021	1	1	1	1	1	1	1	0	1	1	0	0	1	0	0	1	1	1	1	1	1	0	0	0	1	1	1	19/32

*Note*: Assessment of the risk of bias using Downs and Black checklist (Downs & Black, 1998). The Downs and Black checklist have a maximum score of 32 points divided into five domains, as follows: (1) Reporting, items 1–10; (2) External validity, items 11–13; (3) Internal validity ‐ bias, items 14–20; (4) Internal validity—confounding (bias), items 21–26; (5) Power, item 27.

### 3.4 Effects of exercise on nucleotidases

Among the selected studies, the main effects of different exercise interventions on nucleotidase activities and expression are presented in Table 2. Nine studies (Coppola et al., 2005; Dorneles et al., 2019; Karabulut et al., 2011; Kirby et al., 2012; Mânica et al., 2020; Miron et al., 2019; Moritz et al., 2017; Moritz et al., 2021; Yegutkin et al., 2007) investigated the outcomes of an acute exercise intervention on nucleotidases behavior in healthy individuals. Coppola et al. (Coppola et al., 2005) demonstrated that acute strenuous exercise decreased NTPDase1/CD39 expression in platelets, in sedentary (*p* < 0.01) and physically active (*p* < 0.01) subjects; however, the expression of NTPDase1/CD39 increased in B‐lymphocytes (sedentary *p* < 0.005; physically active *p* < 0.005).

Yegutkin et al. (Yegutkin et al., 2007) first showed that maximal and submaximal acute exercise increased plasma ATP (*p* < 0.05) and ADP (*p* < 0.05) levels in trained subjects, and remained increased for 10 min after exercise, while AMP (*p* < 0.05) levels increased at maximal intensity exercise (*p* < 0.05). Furthermore, the activities of soluble NPP and NTPDase increased in plasma during submaximal and maximal exercise (*p* < 0.05); however, soluble NTPDase activities remained increased at 10 min after exercise (*p* < 0.05). The same study demonstrated that the incremental test, performed by trained individuals, increased ADP hydrolysis during (*p* < 0.05) and after the test (*p* < 0.05), reaching peak values after 10 min (*p* < 0.05) of exercise and returning to resting levels after 30 min. The pattern of nucleotide hydrolysis was similar in arterial and venous blood. A third assay was performed in the same study with healthy sedentary subjects that performed an incremental test, showing increased levels of plasma ATP at maximal intensity (*p* < 0.05). Moreover, soluble NTPDase and NPP activities transiently increased during maximal exercise in the serum of sedentary individuals (*p* < 0.05), returning to basal levels during the recovery period.

Karabulut et al. (Karabulut et al., 2011) investigated the effect of acute exercise (20‐meter shuttle run test) on ADA activity in the blood plasma of sedentary healthy men and women. This study showed an increased ADA activity in women (*p* = 0.002) at post‐exercise, but no effect of exercise in men (*p* = 0.630). The study performed by Kirby et al. (Kirby et al., 2012) assessed ATP hydrolysis in whole blood and the levels of plasma ATP during graded‐intensity handgrip exercise in healthy young and older adults. The data showed a similar increase in ATP hydrolysis of young and older adults (*p* < 0.05), combined with higher levels of plasma ATP in young adults compared to older adults (*p* < 0.05) during acute resistance exercise.

Moritz et al. (Moritz et al., 2017) analyzed the influence of a moderate acute aerobic exercise session on nucleotidase activities, and nucleotides and nucleosides levels in the blood serum of healthy sedentary male individuals. The results showed increased activities of soluble NTPDases (*p* < 0.05), 5’‐NT/CD73 (*p* < 0.05), and NPP (*p* < 0.05) at post‐exercise, in association with reduced serum levels of ATP (*p* < 0.05), ADP (*p* < 0.05), and increased serum levels of ADO (*p* < 0.05) and INO (*p* < 0.05). In more recent work (Moritz et al., 2021), our research group also assessed the effects of an acute aerobic exercise session on nucleotidase activities, and ATP metabolism in the blood plasma of sedentary, overweight, and physically active male individuals. This data demonstrated increased activities of soluble NTPDases (*p* < 0.05), 5’‐NT/CD73 (*p* < 0.05), and NPP (*p* < 0.05) immediately post‐exercise, while the activity of 5’‐NT/CD73 (*p* < 0.05) remain increased until 1 h after the exercise session in all groups. Moreover, the levels of ATP (*p* < 0.05) and ADP (*p* < 0.05) in plasma remain decreased until 1 h after the exercise, while the levels of ADO (*p* < 0.05) remain augmented until 1 h after exercise in all groups. Additionally, the analyses detected a positive correlation between the activity of 5’‐NT/CD73, adenosine levels, cardiorespiratory fitness, tumor necrosis factor α (TNF‐α), and interleukin‐8 (IL‐8).

Two studies (Dorneles et al., 2019; Miron et al., 2019) evaluated the effects of acute high‐intensity interval exercise protocols (HIIE), with a few differences, on nucleotidases function in the platelets and lymphocytes of healthy male subjects. Miron et al. (Miron et al., 2019) showed that an acute protocol of HIIE, when applied to semi‐professional athletes did not significantly change ATP, ADP and AMP hydrolysis and NTPDase1/CD39 expression in lymphocytes at post‐exercise and after 30 min. However, ADA activity in lymphocytes increased post‐exercise (*p* < 0.05), and remained higher after 30 min (*p* < 0.05). In contrast, ATP and ADP hydrolysis in platelets decreased at post‐exercise (*p* < 0.05) and returned to basal levels after 30 min. On the other hand, ADA activity increased post‐exercise (*p* < 0.05) and returned to basal levels after 30 min.

Dorneles et al. (Dorneles et al., 2019) assessed the impact of HIIE on NTPDase1/CD39 and 5’‐NT/CD73 expression in the CD4^+^CD25^−^ and CD4^+^CD25^+^ T cells of low fitness and high fitness healthy male subjects. The expression of NTPDase1/CD39 increased in CD4^+^CD25^−^ and CD4^+^CD25^+^ T cells in the low fitness (*p* = 0.021 and *p* = 0.03, respectively) and high fitness (*p* = 0.025 and *p* = 0.39, respectively) groups after the acute session of HIIE. The values increased further after 1 h in the CD4 + CD25‐ T cells of the in low fitness group (*p* = 0.042) and in the CD4^+^CD25^+^ T cells of the high and low fitness groups (*p* = 0.035 and *p* = 0.033, respectively). The expression of 5’‐NT/CD73 enhanced on CD4^+^CD25^+^ T cells post‐exercise in both groups (*p* = 0.01), remaining elevated at 1 h after the HIIE session in low fitness (*p* = 0.042) and high fitness group (*p* = 0.023).

Mânica et al. (Mânica et al., 2020) investigated the acute effects of low‐intensity aerobic exercise, high‐intensity aerobic exercise, and low‐intensity aerobic exercise with blood flow restriction on the nucleotidases activities in lymphocytes of hypertensive patients. The results demonstrated a similar increase in the ATP (*p* < 0.05) and ADP (*p* < 0.05) hydrolysis 30 min after high‐intensity aerobic exercise and low‐intensity aerobic with blood flow restriction. However, ADA (*p* < 0.05) activity was reduced only 30 min after high‐intensity aerobic exercise. Additionally, no effect was detected after acute low‐intensity aerobic exercise.

Martins et al. (Martins, Bagatini, Cardoso, Zanini, Abdalla, Baldissarelli, Dalenogare, dos Santos, et al., 2016; Martins, Bagatini, Cardoso, Zanini, Abdalla, Baldissarelli, Dalenogare, Farinha, et al., 2016) assessed the impact of 30 weeks of concurrent training on the nucleotidase activities of platelets and lymphocytes of metabolic syndrome patients. The first study (Martins, Bagatini, Cardoso, Zanini, Abdalla, Baldissarelli, Dalenogare, Farinha, et al., 2016) showed that, under pre‐training conditions, platelets of metabolic syndrome patients presented an increase in NTPDase (*p* < 0.001), 5’‐NT/CD73 (*p* < 0.001) and NPP (*p* < 0.001) activities, and a decrease in ADA (*p* < 0.001) activity, compared to the control group. After the training period, the NTPDase (*p* < 0.001), 5’‐NT/CD73 (*p* < 0.001) and NPP (*p* < 0.001) activities decreased, and ADA (*p* < 0.001) activity increased, compared to the pre‐training condition, resembling the control group. The second study (Martins, Bagatini, Cardoso, Zanini, Abdalla, Baldissarelli, Dalenogare, dos Santos, et al., 2016) showed that, in the pre‐training condition, lymphocytes of metabolic syndrome patients demonstrated an increase in ATP, ADP hydrolysis (*p* < 0.001), and a decrease in ADA activity (*p* < 0.001), compared to the control group. After 30 weeks of training ATP and ADP hydrolysis (*p* < 0.05) decreased and ADA (*p* < 0.05) activity increased, compared to the pre‐training condition, demonstrating similar levels to those of the control group.

Finally, Silveira et al. (da Silveira et al., 2018) evaluated the effects of 8 weeks of resistance training on nucleotidase activities in the platelets of chronic kidney disease patients. ATP (*p* < 0.0001) and ADP (*p* < 0.0001) hydrolysis decreased after 8 weeks of training, while no significant differences were observed in AMP hydrolysis and ADA activity. Moreover, Lammers et al. (Lammers et al., 2020) evaluated the effects of 27 weeks of resistance training on the nucleotidase activities, NTPDase1/CD39 and NTPDase2/CD39L1 expression in lymphocytes and serum levels of ATP of hypertensive and normotensive subjects. Firstly, under pre‐training conditions, lymphocytes of hypertensive subjects showed an increase in ATP (*p* < 0.05), ADP (*p* < 0.05) hydrolysis, ADA (*p* < 0.05) activity, and serum levels of ATP (*p* < 0.05) compared to the normotensive group. After the training period, ATP (*p* < 0.05), ADP (*p* < 0.05) hydrolysis, and ADA (*p* < 0.05) activity decreased, as well as the serum levels of ATP (*p* < 0.05) in the hypertensive group compared to the pre‐training condition. In contrast, the expression of NTPDase1/CD39 and NTPDase2/CD39L1 did not significantly change in lymphocytes of hypertensive or normotensive subjects after the training protocol.

## 4 DISCUSSION

This systematic review was performed to evaluate the effects of different exercise modalities on purinergic enzyme activities and expression. The findings of the current review suggest that different exercise modalities modulate the activities and expression of NTPDases, NPPs, 5’‐NT/CD73, and ADA. These data indicate a novel role for purinergic signaling in linking exercise biochemistry and physiology.

As mentioned, acute and chronic exercise promotes multiple systemic adaptations (Heinonen et al., 2014). Thus, it is reasonable to expect that nucleotidases responses to exercise follow some basic principles in response to the type of exercise, frequency, intensity, duration, volume, and biological individuality (American College of Sports Medicine et al., 2018). The results of this systematic review suggest that acute exercise increases nucleotidase responses, at least in some pathways, in healthy subjects. Copolla et al. (Coppola et al., 2005) and Dorneles et al. (Dorneles et al., 2019) observed an increase in NTPDase1/CD39 expression in B‐lymphocytes, CD4^+^CD25^−,^ and CD4^+^CD25^+^ T cells in healthy sedentary and physically active males after maximal exercise test and HIIE acute session. Yegutkin et al., 2007; Kirby et al., 2012; Moritz et al., 2017; Moritz et al., 2021 also observed an increase in nucleotidase activities in response to different exercise protocols, applied to trained, and sedentary healthy individuals. Additionally, these studies demonstrated that exercise was able to modify, at least transiently, purine levels in blood plasma and serum.

The data cited above is related to the inflammatory and coagulatory effects that are triggered by exercise. It is well known that moderate and intense acute exercise promote inflammatory and coagulatory responses, increasing levels of white blood cells (WBC), interleukin‐6 (IL‐6), IL‐8, interleukin‐10 (IL‐10), and C‐reactive protein (CRP) as well as elevating platelet count, platelet sensitivity to ADP, and collagen‐induced aggregation, prothrombin activation peptide 1.2 (F1.2) and thrombin/antithrombin complexes (TAT) (Cerqueira et al., 2020; Lippi & Maffulli, 2009; Posthuma et al., 2015). These biological processes are closely related to purinergic signaling, highlighting their responses in inflammatory (Cekic & Linden, 2016) and coagulatory (Anyanwu et al., 2019; Atkinson et al., 2006; Hechler & Gachet, 2015) function.

Extracellular ATP has a well‐established pro‐inflammatory effect, promoting chemotaxis, oxygen free radical generation, and IL‐6 and TNF‐α production, whereas ADO has an anti‐inflammatory effect, decreases superoxide anion production, and pro‐inflammatory cytokine release, inhibits apoptosis and increases IL‐10 release (Faas et al., 2017; Schetinger et al., 2007). Additionally, ATP is an inhibitor of ADP‐induced platelet aggregation (Coade & Pearson, 1989) and ADO (produced via 5’‐NT/CD73) is an important vasodilator and inhibitor of platelet aggregation (Birk et al., 2002). The release of ATP by erythrocytes is a relevant mechanism of vasodilatation via endothelial P2Y_1/2/4_ receptors in response to hypoxia, shear stress, and low pH (Ellsworth et al., 2016). Thus, we can assume that different exercise protocols, when applied in healthy sedentary and physically active subjects, modulate the activities, and expression of nucleotidases to balance nucleotides and nucleosides levels, contributing to biochemical and physiological responses to exercise.

Significant advances were made by early studies related to purine metabolism and exercise; however, these studies did not focus on nucleotidase functionality. Hellsten‐Westing et al., 1993 showed a reduction in the plasma levels of hypoxanthine (HX) and uric acid (UA) after 6 weeks of HIIE training in healthy, physically active males. Kinugawa et al., 2006 reported increased levels of ADO in NYHA class III chronic heart failure patients, compared to less severe patients and healthy controls at basal and post‐exercise conditions. The increased levels of plasma adenosine adjusted to exercise the workload may be an adaptive response in cardiac patients. Additionally, it is reasonable to assume that severe cardiac patients present higher levels of ADO, due to its cardioprotective effects such as vasodilatation, reduction of contractility and heart rate, and enhancement of O_2_ and substrate delivery (Headrick et al., 2011). Recently, Zarębska et al., 2018 demonstrated plasma ATP, ADP, and AMP concentrations during resting, incremental test, and recovery period in highly trained male athletes and physically active men.

Despite the presence of nucleotidases on hematopoietic cells, in their soluble form and attached to extracellular vesicles, endothelial nucleotidases such as NTPDase1/CD39, NTPDase2/CD39L1, and 5’‐NT/CD73 are the main regulators of nucleotides and nucleosides levels in the blood (Deaglio & Robson, 2011; Yegutkin et al., 2003). Furthermore, multiple pathways of nucleotide inactivation and nucleotide resynthesis are involved in the purinergic signaling. In this context, a network of enzymes may influence the extracellular levels of nucleotides and nucleosides that modulate inflammatory, thrombotic, and vascular responses (Yegutkin, 2014; Yegutkin et al., 2002).

Two other of the selected studies (Martins, Bagatini, Cardoso, Zanini, Abdalla, Baldissarelli, Dalenogare, dos Santos, et al., 2016; Martins, Bagatini, Cardoso, Zanini, Abdalla, Baldissarelli, Dalenogare, Farinha, et al., 2016) evaluated the effects of moderate concurrent training for 30 weeks on the platelets and lymphocytes of metabolic syndrome patients. Additionally, Lammers et al., 2020 investigated the impacts of resistance training for 27 weeks in lymphocytes of hypertensive patients. These studies demonstrated that, under pre‐training conditions, metabolic syndrome, and hypertensive patients present an increase in NTPDase, NPP, 5’‐NT/CD73, and ADA activity. However, ADA activity decrease under pre‐training conditions in platelets and lymphocytes of metabolic syndrome patients. Such results are in agreement with previous studies that demonstrated an increase in nucleotidase activities in conditions of diabetes, hypercholesterolemia, hyperglycemia, and hypertension (Cardoso et al., 2015; Lunkes et al., 2003; Lunkes et al., 2008; Medeiros Frescura Duarte et al., 2007). Martins, Bagatini, Cardoso, Zanini, Abdalla, Baldissarelli, Dalenogare, dos Santos, et al., 2016; Martins, Bagatini, Cardoso, Zanini, Abdalla, Baldissarelli, Dalenogare, Farinha, et al., 2016 and Lammers et al., 2020 showed that concurrent training for 30 weeks and resistance training for 27 weeks, respectively, are capable of reversing and normalizing the nucleotidase activities of platelets and lymphocytes to activities similar to those of healthy subjects. These data can be associated with improved cardiovascular (Laukkanen et al., 2001) and inflammatory (Cerqueira et al., 2020) parameters promoted by chronic exercise, since long‐term exercise reduces glycemia levels, blood pressure, high‐density lipoprotein (HDL), low‐density lipoprotein (LDL), cholesterol, triglycerides, pro‐inflammatory markers, and increases anti‐inflammatory markers.

Correlation analysis showed a synergic function between NTPDases, NPP, 5’‐NT/CD73, ADA activities and levels of glucose, HDL, triglycerides, peak oxygen uptake (VO_2peak_) mean platelet volume, platelet aggregation, CRP, waist circumference, and blood pressure in lymphocytes and/or platelets (Cardoso et al., 2015; Lammers et al., 2020; Lunkes et al., 2008; Martins, Bagatini, Cardoso, Zanini, Abdalla, Baldissarelli, Dalenogare, dos Santos, et al., 2016; Martins, Bagatini, Cardoso, Zanini, Abdalla, Baldissarelli, Dalenogare, Farinha, et al., 2016; Moritz et al., 2021). These data suggest that, since long‐term exercise is able to modify cardiometabolic parameters, the nucleotidase activities could change in relation to them.

Previous studies (Fürstenau et al., 2008; Köhler et al., 2007; Marcus et al., 2005; Moritz et al., 2017) suggest a protective role of purinergic signaling against stressful or disease conditions. Moreover, a compensatory physiological mechanism has also been suggested, in function of the ability of nucleotidase activities to decrease ATP and ADP levels (pro‐inflammatory and pro‐thrombotic molecules, respectively), and increase ADO and INO levels, both cytoprotective molecules (Anyanwu et al., 2019; Cardoso et al., 2012; Fürstenau et al., 2008; Martins, Bagatini, Cardoso, Zanini, Abdalla, Baldissarelli, Dalenogare, dos Santos, et al., 2016; Schetinger et al., 2007).

## 5 STUDY STRENGTHS AND LIMITATIONS

This systematic review is the first to highlight the effects of exercise on nucleotidase functionality in healthy and unhealthy subjects using a high‐quality methodological approach in accordance with PRISMA statement. One of the limitations of our work was due to the methodological diversity in the evaluation of nucleotidase activities and expression applied among the included studies, such as high‐performance liquid chromatography (HPLC), flow cytometry, thin layer chromatography (TLC), and spectrometric assay. However, the pattern of nucleotidases responses to different, acute or chronic, exercise modalities was quantified as demonstrated in Table 2.

Additional limitations are present in our study and should be pointed out: (1) only one randomized clinical trial (Mânica et al., 2020) was found in our search; (2) there wre methodological differences in the exercise protocols used in selected studies (since there are a limited number of published studies on this research subject, it was impossible to restrict the exercise method or type); (3) due to the high heterogeneity of data and subjects included in this systematic review, it was not possible to perform a meta‐analysis; (4) taking into account the quality assessment, some studies were considered to be methodologically limited; (5) we did not include gray literature in the search strategy.

## 6 CONCLUSIONS

Our results suggest that acute and chronic protocols of exercise induce at least transitory adaptations in NTPDase, NPP, 5’‐NT/CD73, and ADA activities and expression in different biological samples. These nucleotidases adaptations modified nucleotides and nucleosides levels, possibly modulating coagulation, inflammation, and vascular activity associated with the exercise response. Moreover, since long‐term exercise induces improvements in cardiometabolic and inflammatory parameters, purinergic enzymes functionality may be related to these markers. The data from this systematic review suggest novel biochemistry and physiological adaptations induced by exercise, based on purinergic signaling. Due to the heterogeneity of included studies, some of our results should be treated carefully. Therefore, further studies with high methodological quality are necessary to support the concept of a purinergic pathway as a mechanism of physiological adaptation promoted by different exercise strategies, as well as clinical relevance.

## AUTHOR CONTRIBUTIONS

C.E.J.M., F.F., A.M.O.B., and A.R.‐O. contributed to the conception and design of the work. C.E.J.M, A.F.V., and D.M.M. performed the data collection and analysis. C.E.J.M. wrote the manuscript. A.M.O.B., F.F. and, A.R.‐O. edited and revised the manuscript. All authors approved the final version of the manuscript and agree to be accountable for all aspects of the work in ensuring that questions related to the accuracy or integrity of any part of the work are appropriately investigated and resolved. All persons designated as authors qualify for authorship, and all those who qualify for authorship are listed.

## FUNDING INFORMATION

C.E.J.M., A.F.V., and D.M.M. were supported by fellowships from the Coordination for the Improvement of Higher Education Personnel (CAPES, Brazil). A.R.‐O. and A.M.O.B. are Research Productivity Fellows of the Brazilian National Council for Scientific and Technological Development (CNPq, Brazil). We thank Fundação de Amparo à Pesquisa do Estado do RS (FAPERGS), Programa Pesquisador Gaúcho (project number 17/2551‐0000 970‐3), Brazil, for the financial support of this work.

## CONFLICT OF INTEREST

The authors declare no conflict of interest.

## ETHICS STATEMENT

This work does not contain any studies with human participants or animals performed by any of the authors.

## REGISTRATION NUMBER

CRD42019110593.

## Supporting information


Table S1
