# Supplementary material for: Effects of physical exercise on the functionality of human nucleotidases: A systematic review
Source: Physiol Rep. 2022 Sep 18;10(18):e15464. doi: 10.14814/phy2.15464 (PMC9483616; doi:10.14814/phy2.15464)
Supplement: Supplementary file 1 — Table S1 [file PHY2-10-e15464-s001.docx]

Supplementary Table 1. Search strategy used in PubMed

| Step | Search terms |
| --- | --- |
| #1 | "Exercise"[Mesh] OR "Exercises" OR "Physical Activity" OR “Activities, Physical” OR “Activity, Physical” OR “Physical Activities” OR “Exercise, Physical” OR “Exercises, Physical” OR “Physical Exercise” OR “Physical Exercises” OR “Acute Exercise” OR “Acute Exercises” OR “Exercise, Acute” OR “Exercises, Acute” OR “Exercise, Isometric” OR “Exercises, Isometric” OR “Isometric Exercises” OR “Isometric Exercise” OR “Exercise, Aerobic” OR “Aerobic Exercise” OR “Aerobic Exercises” OR “Exercises, Aerobic” OR “Exercise Training” OR “Exercise Trainings” OR “Training, Exercise” OR “Trainings, Exercise” |
| #2 | “Nucleotidases”[Mesh] OR “Apyrase” OR “Phosphohydrolase, ADP” OR “ADPase” OR “ATP-ADPase” OR “ATP ADPase” OR “ATP-Diphosphatase” OR “ATP Diphosphatase” OR “ATP Diphosphohydrolase” OR “Diphosphohydrolase, ATP” OR “Adenosine Diphosphatase” OR “Diphosphatase, Adenosine” OR “ENTPD1 protein, human” OR “ATPDase, human” OR “CD39 antigen, human” OR “ectonucleoside triphosphate diphosphohydrolase 1, human” OR “NTPDase-1, human” OR “ectoATPase” OR “ecto-adenosine triphosphatase” OR “ecto-atpase” OR “ATPase, ecto” OR “NTPDase2” OR “CD39L1 protein, human” OR “ENTPD2 protein, human” OR “NTPDase-3, human” OR “ectonucleoside triphosphate diphosphohydrolase 3, human” OR “CD39-like 3 protein, human” OR “CD39L3 protein, human” OR “ecto-Mg-ATPase” OR “Myoglein” OR “ecto-nucleotidase” OR “CD39 antigen” OR “ecto-apyrase” OR “NTPDase1” OR “ectoADPase” OR “ectonucleoside triphosphate diphosphohydrolase 1” OR “NTPDase-1” OR “ecto-ADPase” OR “ecto-ATP diphosphohydrolases” OR "CD39" OR “5'-Nucleotidase” OR “5' Nucleotidase” OR “5'-Nucleotidase Phosphoribolase” OR “5' Nucleotidase Phosphoribolase” OR “Adenylate Phosphatase” OR “AMP Phosphatase” OR “Cytidylate Phosphatase” OR “Ecto-5'-Nucleotidase” OR “Ecto 5' Nucleotidase” OR “IMP Nucleotidase” OR “CD73 Antigens” OR “IMPase” OR “Inosinate Phosphatase” OR “Pyrimidine 5'-Nucleotidase” OR “Pyrimidine 5' Nucleotidase” OR “Thymidine Phosphatase” OR “Uridylate 5'-Nucleotidase” OR “Uridylate 5' Nucleotidase” OR “Antigen, CD73” OR “CD73 Antigen” OR “Antigens, CD73” OR “5'-AMP Nucleotidase” OR “5' AMP Nucleotidase” OR “IMP Phosphatase” OR “ectonucleotide pyrophosphatase phosphodiesterase 1” OR “nucleotide pyrophosphatase-alkaline phosphodiesterase I” OR “ecto-nucleotide pyrophosphatase phosphodiesterase 1” OR "CD73" OR “glycoprotein PC-1” OR “plasma cell membrane glycoprotein PC-1” OR “Enpp1 protein, zebrafish” OR “MAPF protein, Bos taurus" OR “major acidic fibroblast growth factor-stimulated phosphoprotein, Bos taurus” OR “major aFGF-stimulated phosphoprotein, Bos taurus” OR “ENPP1 protein, rat” OR “ENPP1 protein, human” OR “NPP1 protein, human” OR "alkaline phosphodiesterase 1, human" OR "PC-1 glycoprotein, human" OR "plasma-cell membrane glycoprotein 1, human" OR "ectonucleotide pyrophosphatase-phosphodiesterase 1, human" OR "ENPP1 protein, mouse" OR "nucleotide pyrophosphatase - phosphodiesterase I" OR "NPP-PDE" OR “Adenosine Deaminase” OR “Deaminase, Adenosine” OR “Adenosine Aminohydrolase” OR “Aminohydrolase, Adenosine” OR "ADA" |
| #3 | “Adenosine Triphosphate”[Mesh] OR “ATP” OR “Adenylpyrophosphate” OR “Adenosine Triphosphate, Magnesium Salt” OR “Magnesium Adenosine Triphosphate” OR “MgATP” OR “Adenosine Triphosphate, Manganese Salt” OR “MnATP” OR “Manganese Adenosine Triphosphate” OR “Atriphos” OR “Adenosine Triphosphate, Chromium Salt” OR “CrATP” OR “Cr(H2O)4 ATP” OR “Chromium Adenosine Triphosphate” OR “Adenosine Triphosphate, Calcium Salt” OR “CaATP” OR “Adenosine Triphosphate, Chromium Ammonium Salt” OR “Adenosine Triphosphate, Magnesium Chloride” OR “ATP-MgCl2” OR “ATP MgCl2” OR “Striadyne” OR “Adenosine Diphosphate”[Mesh] OR “Diphosphate, Adenosine” OR “ADP” OR “Adenosine Pyrophosphate” OR “Pyrophosphate, Adenosine” OR “Adenosine 5'-Pyrophosphate” OR “5'-Pyrophosphate, Adenosine” OR “Adenosine 5' Pyrophosphate” OR “Magnesium ADP” OR “ADP, Magnesium” OR “MgADP” OR “Adenosine Monophosphate”[Mesh] OR “AMP” OR “Adenosine 5'-Phosphate” OR “5'-Phosphate, Adenosine” OR “Adenosine 5' Phosphate” OR “Adenosine Phosphate Dipotassium” OR “Dipotassium, Adenosine Phosphate” OR “Phosphate Dipotassium, Adenosine” OR “Disodium, Adenosine Phosphate”) OR “Phosphate Disodium, Adenosine” OR “Adenosine 2'-Phosphate” OR “Adenosine 2' Phosphate” OR “2'-Adenylic Acid” OR “2' Adenylic Acid” OR “Acid, 2'-Adenylic” OR “2'-Adenosine Monophosphate” OR “2' Adenosine Monophosphate” OR “Monophosphate, 2'-Adenosine” OR “Adenylic Acid” OR “2'-AMP” OR “5'-Adenylic Acid” OR “5' Adenylic Acid” OR “Acid, 5'-Adenylic” OR “Adenosine 3'-Phosphate” OR “Adenosine 3' Phosphate” OR “Phosphaden” OR “Adenosine”[Mesh] OR “Adenocard” OR “Adenoscan” OR “Inosine” |
| #4 | #1 AND #2 AND #3 |
